# Synergistic Action of a Microbial-based Biostimulant and a Plant Derived-Protein Hydrolysate Enhances Lettuce Tolerance to Alkalinity and Salinity

**DOI:** 10.3389/fpls.2017.00131

**Published:** 2017-02-07

**Authors:** Youssef Rouphael, Mariateresa Cardarelli, Paolo Bonini, Giuseppe Colla

**Affiliations:** ^1^Department of Agricultural Sciences, University of Naples Federico IIPortici, Italy; ^2^Consiglio per la Ricerca in Agricoltura e l’Analisi dell’Economia Agraria, Centro di Ricerca per lo Studio delle Relazioni tra Pianta e SuoloRome, Italy; ^3^NGA LaboratoryTarragona, Spain; ^4^Department of Agricultural and Forestry Sciences, Tuscia UniversityViterbo, Italy

**Keywords:** antioxidant enzymes, chlorophyll fluorescence, *Lactuca sativa* L., mineral composition, pH level, proline, *Rhizophagus intraradices*, *Trichoderma atroviride*

## Abstract

In the coming years, farmers will have to deal with growing crops under suboptimal conditions dictated by global climate changes. The application of plant biostimulants such as beneficial microorganisms and plant-derived protein hydrolysates (PHs) may represent an interesting approach for increasing crop tolerance to alkalinity and salinity. The current research aimed at elucidating the agronomical, physiological, and biochemical effects as well as the changes in mineral composition of greenhouse lettuce (*Lactuca sativa* L.) either untreated or treated with a microbial-based biostimulant (Tablet) containing *Rhizophagus intraradices* and *Trichoderma atroviride* alone or in combination with a PH. Plants were sprayed with PH at weekly intervals with a solution containing 2.5 ml L^-1^ of PH. Lettuce plants were grown in sand culture and supplied with three nutrient solutions: standard, saline (25 mM NaCl) or alkaline (10 mM NaHCO_3_ + 0.5 g l^-1^ CaCO_3_; pH 8.1). Salt stress triggered a decrease in fresh yield, biomass production, SPAD index, chlorophyll fluorescence, leaf mineral composition and increased leaf proline concentration, without altering antioxidant enzyme activities. The decrease in marketable yield and biomass production under alkali stress was not significant. Irrespective of nutrient solution, the application of Tablet and especially Tablet + PH increased fresh marketable yield, shoot and root dry weight. This was associated with an improvement in SPAD index, *F*_v_/*F*_m_ ratio, CAT and GPX activities and a better nutritional status (higher P, K, and Fe and lower Na with NaCl and higher P and Fe with NaHCO_3_) via an increase of total root length and surface. The combination of microbial biostimulant with foliar application of PH synergistically increased the marketable fresh yield by 15.5 and 46.7% compared to the Tablet-treated and untreated plants, respectively. The improved crop performance of Tablet + PH application was attributed to a better root system architecture (higher total root length and surface), an improved chlorophyll synthesis and an increase in proline accumulation. Combined application of Tablet and PH could represent an effective strategy to minimize alkalinity and salinity stress in a sustainable way.

## Introduction

Salinity and alkalinity are among the major and increasing environmental stresses affecting crop production, particularly in arid and semi-arid areas (Guo et al., 2015). Most of the vegetable crops are salt and alkali sensitive, growing poorly in salinized and alkaline soils (Colla et al., 2010; Rouphael et al., 2010). Soil salinity is mainly due to the accumulation of toxic ions (Na^+^, Cl^-^, and SO_4_^2-^; Colla et al., 2012, 2013a), whereas alkaline soils are generally characterized by high concentrations of bicarbonate (HCO_3_^-^) and carbonate (CO_3_^2-^) as well as high pH and almost no exchangeable H^+^ (Misra and Tyler, 1999; Marschner, 2012). Excessive concentrations of sodium chloride (NaCl) in soil or water can induce several morphological, physiological, and biochemical responses of plants leading to stunted growth and yield (Borgognone et al., 2016; Lucini et al., 2016; Rouphael et al., 2017). This is due to osmotic (i.e., water deficit stress) and ionic (i.e., Na^+^ and Cl^-^) effects on nutrient uptake/translocation and metabolic processes such as nitrogen assimilation, photosynthesis, and protein synthesis (Tester and Davenport, 2003).

Similar to salinity, alkalinity can inhibit crop performance due to the high osmotic potential of the soil. In addition, high alkaline environment in the rhizosphere can reduce or even inhibit the uptake of macro- (H_2_PO_4_^-^, Ca^2+^, and Mg^2+^) (Yang et al., 2007) and micro ions, especially Fe^2+^. Iron deficiency has been often considered as the main factor responsible for leaf chlorosis and yield losses in calcareous soils, since the reduction in leaf iron concentration is accompanied by a significant reduction in chlorophyll synthesis and fluorescence as well as limitation in photosynthetic CO_2_ fixation (Molassiotis et al., 2006; Cardarelli et al., 2010). Under abiotic stress conditions including salinity and alkalinity, plants have developed different tolerance and protective mechanisms to cope with the formation of reactive oxygen species (ROS) that cause oxidative stress (Mittler, 2002). The antioxidant defense system includes both non-enzymatic low molecular-weight antioxidants (ascorbate, carotenoids, glutathione, and α-tocopherol) as well as antioxidant enzymes (ascorbate peroxidase, catalase, and guaiacol peroxidase) (Noctor and Foyer, 1998; Kumar et al., 2015a,b).

A sustainable tool and a meaningful approach to counteract salt and alkaline stresses would be the use of plant biostimulants, which are gaining interest worldwide (Colla and Rouphael, 2015; Garcia-Mina and Hadavi, 2016). The term plant biostimulants (PBs) refers to ‘*any substance or microorganism applied to plants with the aim to enhance nutrition efficiency, abiotic stress tolerance and/or crop quality traits, regardless of its nutrient content*’ (du Jardin, 2015). According to Colla et al. (2015a) protein hydrolysates (PHs; i.e., mixtures of polypeptides, oligopeptides, and amino acids) have gained prominence as PBs in vegetable crop production. The leaf and/or root application of PHs on vegetables has been reported to improve tolerance to adverse soil conditions and environmental stresses. These include thermal stress (Botta, 2013), alkalinity (Cerdán et al., 2009), drought (Petrozza et al., 2014), salinity (Lucini et al., 2015), and nutrient deficiency (Colla et al., 2013b, 2014). The positive effects exerted by PHs to stresses have often been associated to oxidative stress mitigation, osmotic adjustment, and to changes in hormone networks (Colla et al., 2015a; Lucini et al., 2015).

Microbial biostimulants such as arbuscular mycorrhizal fungi (AMF) and *Trichoderma* spp. are also considered promising tools to overcome the limitations of salinity and alkalinity on crop growth and productivity. Many AMF and *Trichoderma* spp. strains can enhance vegetable tolerance to abiotic stresses by increasing nutrient uptake through greater effective root area (for AMF) and better solubilization of micronutrients (for *Trichoderma* spp.), and also throughout the production into the rhizosphere of small peptides, volatiles and metabolites with hormone activities such as indole-3-acetic acid or auxin analogs (for *Trichoderma* spp.) (Giovannetti et al., 2001; López-Bucio et al., 2015; Rouphael et al., 2015a,b). Recent research showed that co-inoculation of AMF (*Rhizophagus intraradices*) and *Trichoderma atroviride* at transplanting increased plant growth of several vegetable crops under non-stress conditions (Colla et al., 2015b). Nevertheless, nothing is known about the combined use of *R. intraradices* and *T. atroviride* as well as the synergetic potential of PH and microbial biostimulants under conditions of salinity and alkalinity. Therefore, we hypothesized that the application of a plant derived-PH and a microbial based biostimulant containing both *R. intraradices* and *T. atroviride* will further mitigate negative effects of salinity and alkalinity and enhance growth of lettuce (*Lactuca sativa* L.) plants.

To verify the above hypothesis, a greenhouse experiment was conducted to assess the influence of a microbial based biostimulant alone or in combination with a plant derived-PH on lettuce tolerance to salinity and alkalinity, and to elucidate the physiological, biochemical and compositional changes mediated by biostimulant application(s) under salt and alkaline stress conditions.

## Materials and Methods

### Experimental Design and Plant Material

A greenhouse experiment was conducted at the Tuscia University, Viterbo central Italy using a 3 × 3 factorial scheme and completely randomized design with four replicates per treatment, amounting to a total of 36 experimental unit plots with 10 plants each (*n* = 360 plants). The treatments used were three nutrient solutions (standard, alkaline, or saline) and three biostimulant applications (control, microbial inoculum, or microbial inoculum plus plant-derived PH).

Seeds of *L. sativa* L. (cv. Meraviglia d’Inverno; La Semiorto Sementi, Sarno, Italy), were germinated in vermiculite on 29 August 2014. Lettuce seedlings were transplanted 20 days after sowing (18 September), into plastic pots (diameter 14 cm, height 12 cm) containing 1.5 L of quartziferous sand. Pots were disposed in single rows at a plant density of 11 plants m^-2^. Plants were grown under natural light conditions. Inside the greenhouse, the air daily temperature was always maintained below 27°C. Night temperature was always higher than 15°C, while the mean day/night relative humidity was between 55 and 80%.

### Biostimulant Application

The microbial-based biostimulant was a tablet (‘Click Horto’, Italpollina S.p.a., Rivoli Veronese, Italy) containing 200 spores/tablet of *R. intraradices* BEG72 plus 4.5 × 10^7^ CFU/tablet of *T. atroviride* MUCL45632. The microbial-based biostimulant was applied before seedlings transplantation by placing one tablet per pot under the lettuce transplant roots (Colla et al., 2015b).

The commercial plant-derived PH ‘Trainer’ (Italpollina S.p.a, Rivoli Veronese, Italy) was also used in the current study. ‘Trainer’ is a commercial biostimulant with 31% of amino acids and soluble peptides obtained through an advanced enzymatic hydrolysis of proteins from legume seeds (Colla et al., 2014). The treated lettuce plants were uniformly sprayed four times during the growing cycle at weekly intervals with a solution containing 2.5 mL L^-1^ using a 10-L stainless steel sprayer. Foliar application was initiated 6 days after transplanting (DAT) (24 September).

### Nutrient Solution Management, Salinity and Bicarbonate Application

The standard nutrient solution was a modified Hoagland formulation with a composition of (in mM): 8.0 N-NO_3_, 1.0 S, 0.7 P, 2.5 K, 3.0 Ca, 0.7 Mg, 1 N-NH_4_, 1 Na, 1 Cl, and (in μM) 20 Fe, 9 Mn, 0.3 Cu, 1.6 Zn, 20 B, and 0.3 Mo, with an electrical conductivity (EC) of 1.2 dS m^-1^. The saline nutrient solution had the same standard composition plus an additional 24 mM NaCl, giving and EC values of 3.5 dS m^-1^ and a pH of 6.0 ± 0.2. The alkaline nutrient solution also had the same composition of the basic solution plus 10 mM of sodium bicarbonate (NaHCO_3_) and 0.5 g L^-1^ of calcium carbonate (CaCO_3_^-^). Both NaHCO_3_ and CaCO_3_^-^ were added to the nutrient solution to simulate the effect of alkalinity. The pH of the alkaline nutrient solution was 8.1. Both saline and alkaline treatments were initialized 5 DAT.

The nutrient solution was pumped from independent tanks and delivered through a drip-irrigation system at a rate of 2 L min^-1^. Irrigation scheduling was controlled by low-tension tensiometers (LT-Irrometer, USA), which controlled irrigation based on substrate matric potential (Norrie et al., 1994). All tensiometers were connected to an electronic data logger that managed the start (-10 kPa) and the end (-1 kPa) of the irrigation cycle. These two thresholds correspond to the maximum and minimum tension set points, respectively, widely adopted for the major part of the substrates (Rouphael and Colla, 2009). The length of the irrigation cycles was modified to obtain draining of 30% of the nutrient solution from the pots (Rouphael et al., 2006).

### Yield Assessment, Biomass Production, and Root Characteristics

At final harvest (36 DAT), eight lettuce plants per plot were separated into shoots to determine marketable fresh yield, and roots and their tissues were dried in a forced-air oven at 80°C for 72 h for biomass determination. The dried biomass was stored for mineral analysis, while three fresh leaves were collected and instantly frozen in liquid nitrogen and stored at -80°C for later antioxidant enzyme activity and proline analyses.

For the root architecture determination, four lettuce plants per plot were selected. The whole root system was collected by removing the plastic pots. The samples were submerged in bowls filled with deionized water for 30 min. The root system was smoothly washed to eliminate the sand particles. The determination of the root system characteristics was done using WinRHIZO Pro (Regent Instruments Inc., Canada), connected to an image analysis STD 4800 scanner. The roots were arranged in a 20 cm wide and 30 cm long acrylic box filled with 1 cm deionized water. Three-dimensional images were acquired. The total root length (cm plant^-1^), the root diameter (mm) and total root surface (m^2^ plant^-1^) were determined.

### Arbuscular Mycorrhizal Fungi Root Colonization and Quantification of *Trichoderma*

The root colonization by arbuscular mycorrhizal (AM) fungi was determined at the end of the experiment on the same lettuce plants sampled for root measurements. Root samples were cleared with 10% potassium hydroxide (KOH), stained with 0.05% trypan blue in lactophenol as reported by Phillips and Hayman (1970), and microscopically examined for AM fungi colonization by assessing the percentage of root segments containing arbuscules and vesicles using a gridline intercept method (Giovannetti and Mosse, 1980).

The quantification of *Trichoderma* was conducted using the serial plating soil dilution on a *Trichoderma*-selective agar medium as described by Elad et al. (1981). Each root-substrate sample (10 g) was suspended in sterilized distilled water to give a 1:10 dilution. Serial dilutions were made to 10^-8^. Ten microliter aliquot of each dilution and replicates (three) were spread on the *Trichoderma*-selective agar medium in petri dishes. The petri dishes were then incubated for 3 days, after that fungal colonies of *Trichoderma* were detected and counted and the number of CFU per g of dry soil was calculated (Elad et al., 1981).

### SPAD Index and Maximum Quantum Use Efficiency of PSII Measurements

The Soil Plant Analysis Development (SPAD index), a non-destructive measurement of leaf chlorophyll content was recorded three times during the growing cycle at 13, 20, and 36 DAT. A portable chlorophyll meter SPAD-502 (Konica-Minolta Corporation, Ltd., Osaka, Japan) was used to measure the leaf chlorophyll concentration as a rational unit. Twenty fully expanded leaves per replicate were randomly measured and averaged to a single SPAD value for each treatment.

On the same dates, the maximum quantum use efficiency of PSII in dark-adapted state (*F*_v_/*F*_m_; 20 min) was measured using a chlorophyll fluorometer Handy PEA (Hansatech Instruments Ltd, King’s Lynn, UK) with an excitation source intensity higher than 3000 μmol m^-2^ s^-1^ at the sample surface. The minimal fluorescence intensity (*F*_0_) in a dark-adapted state was measured in the presence of a background weak light signal (about 2–3 μmol photons m^-2^ s^-1^). The maximal fluorescence level in the dark-adapted state (*F*_m_) was induced by 0.8 s saturating light pulse (3000 μmol photons m^-2^ s^-1^). The *F*_v_/*F*_m_ was calculated as (*F*_m_ -*F*_0_)/*F*_m_, as reported by Borgognone et al. (2016).

### Antioxidant Enzyme Analyses

For the enzyme assays, the activities of catalase (CAT, EC 1.11.1.6) and guaiacol peroxidase (GPX, EC 1.11.1.7) were measured on the fully expanded fresh leaves stored at -80°C. Enzyme extractions were performed using a pre-chilled mortar and pestle with two volumes of an ice-cold extraction buffer (0.05 M potassium phosphate buffer, pH 7.0) containing 0.1% (w/v) ascorbic acid, 1% (w/v) polyvinylpolypyrrolidone, 1 mM Na_2_–EDTA and 0.1% (v/v) Triton X-100. After centrifugation (15 000 ×*g*, 30 min, 4°C) the supernatant was set aside for the determination of the enzyme activity and protein content by a spectrophotometer (PerkinElmer, Norwalk, CT, USA).

The CAT activity was measured according to Havir and McHale (1989). Assay mixture (1 ml) contained 0.1 ml of 125 mM H_2_O_2_ and 20 μl of the crude extract in 0.05 M potassium phosphate buffer (pH 7.0). Enzyme activity was evaluated by following the decomposition of H_2_O_2_ at 240 nm for 1 min and calculated using the extinction coefficient (0.036 mM^-1^ cm^-1^). Moreover, the GPX activity was measured in accordance with Chance and Maehly (1955). Assay mixture (1 ml) contained 0.1 ml of 90 mM guaiacol, 0.1 ml of 125 mM H_2_O_2_ and 50 μl of the crude extract in 0.05 M potassium phosphate buffer (pH 7.0). Enzyme activity was evaluated following the increase of absorbance at 470 nm for 40 s due to guaiacol oxidation and calculated using the extinction coefficient (26.6 mM^-1^ cm^-1^). Both CAT and GPX enzyme activities were expressed as mmol mg^-1^ protein min^-1^.

### Proline Analysis

Free proline content expressed as μg g^-1^ fw was determined according to the method of Bates et al. (1973) Approximately 0.5 g of leaf material was homogenized in 10 mL of 30 g L^-1^ sulfosalicylic acid (C7H6O6S; Sigma–Aldrich) and the homogenate was filtered through Whatman No. 2 filter paper (Sigma–Aldrich). Then 2 mL of filtrate was reacted with 2 mL of acid-ninhydrin (1.25 g of ninhydrin in 30 mL of glacial acetic acid and 20 mL of 6 mol L^-1^ phosphoric acid) and 2 mL of glacial acetic acid in a test tube at 100°C for 1 h. The reaction was terminated in an ice bath and then 4 mL of toluene was added and the product of the reaction was extracted by vortex mixing. The absorption of the upper phase was read at 520 nm using toluene as a blank. Proline concentration was calculated using L-proline for the standard curve.

### Analysis of Sodium and Mineral Nutrient Concentrations in Leaf Tissue

The dried leaf tissues were ground in a Wiley mill to pass through a 20-mesh screen, then 0.5 g samples were analyzed for sodium and the following macro- and micronutrients: N, P, K, and Fe. Nitrogen (total N) concentration in leaf tissue was determined after mineralization with sulfuric acid (H_2_SO_4_, 96%, Carlo Erba Reagents, Cornaredo, Milan, Italy) in the presence of potassium sulfate (K_2_SO_4_) and a low concentration of copper (Cu) according to the Kjeldahl method (Bremner, 1965). Phosphorus, K, Fe, and Na were determined by dry ashing at 400°C for 24 h, dissolving the ash in HNO_3_ (1:20 w/v) and assaying the solution obtained using an inductively coupled plasma emission spectrophotometer (ICP Iris, Thermo Optek, Milan, Italy; Karla, 1998).

### Statistical Analysis

Analysis of variance (ANOVA) of the experimental data was performed using the SPSS software package (SPSS 13 for Windows, 2001). When ANOVA indicated that either nutrient solution or biostimulant application treatments or their interaction was significant, mean separation was performed using the Duncan’s multiple range test at *p* = 0.05 on each of the significant variables measured.

## Results

### Arbuscular-Mycorrhizal Root Colonization and *Trichoderma* spp. in the Substrate

The percentage of AM root colonization at the end of the trial was influenced by nutrient solution (N) and biostimulant treatment (B) with significant N × B interaction, whereas the number of *Trichoderma* colonies recovered from the medium was only affected by biostimulant application (**Table 1**). No AM fungi colonization was observed in roots of uninoculated plants. However, when the lettuce was supplied with the microbial biostimulant tablet, the percentage of AM infection varied significantly among plants grown under different nutrient solution and biostimulant treatments. In fact, the highest percentage of root colonization was recorded in both biostimulant treatments (Tablet and Tablet + PH) with standard nutrient solution whereas the lowest values were observed in saline-treated plants. An intermediate percentage of root colonization was observed in alkaline-irrigated plants, although it was not significantly different from the values recorded in plants irrigated with standard and saline solution (**Table 1**). Moreover, irrespective of nutrient solution, the highest number of *Trichoderma* colonies, measured as CFU g^-1^ were recorded in both biostimulant treatments compared to untreated lettuce plants (**Table 1**).

**Table 1:** Analysis of variance and mean comparisons for mycorrhizal root colonization and *Trichoderma* spp. population in roots of lettuce plants grown with different nutrient solutions and biostimulants.

Source of variance	Mycorrhiza root colonization (%)	*Trichoderma* spp. (CFU/g)
Nutrient solution (N)	^∗∗∗^	ns
Biostimulant (B)	^∗∗∗^	^∗∗^
N × B	^∗∗∗^	ns
Nutrient solution		
Standard	21.3	6.3 × 10^4^
Alkaline	16.8	1.6 × 10^4^
Saline	11.8	4.0 × 10^4^
Biostimulant		
No application	0.0	3.9 × 10^2b^
Tablet	24.9	6.3 × 10^4a^
Tablet + PH	25.0	5.5 × 10^4a^
N × B		
Standard solution without biostimulant	0.0^c^	8.7 × 10^2^
Standard solution + Tablet	31.8^a^	1.0 × 10^5^
Standard solution + Tablet + PH	32.1^a^	8.8 × 10^4^
Alkaline solution without biostimulant	0.0^c^	1.1 × 10^2^
Alkaline solution + Tablet	25.6^ab^	2.7 × 10^4^
Alkaline solution + Tablet + PH	24.9^ab^	2.1 × 10^4^
Saline solution without biostimulant	0.0^c^	1.8 × 10^2^
Saline solution + Tablet	17.3^b^	6.2 × 10^4^
Saline solution + Tablet + PH	18.0^b^	5.7 × 10^4^

*ns, ^∗∗^_,_^∗∗∗^ Non-significant or significant at *P* ≤ 0.01, and 0.001, respectively. Different letters within each column indicate significant differences according to Duncan’s multiple-range test (*P* = 0.05). Tablet = microbial-based biostimulant ‘Click Horto’ containing 200 spores/tablet of *Rhizophagus irregularis* BEG72 plus 4.5 × 10^*7*^ CFU/tablet of *Trichoderma atroviride* MUCL45632; PH = plant derived-protein hydrolysate ‘Trainer.’*

### Biomass Production and Root Morphology

The lettuce shoot fresh yield and dry biomass were significantly affected by nutrient solution and biostimulant treatment, with no significant N × B interaction, whereas the root dry weight was highly influenced by N × B interaction (**Table 2**). Irrespective of biostimulant treatment, the shoot fresh yield and dry biomass decreased with increasing salinity in the nutrient solution, with no detrimental effect recorded at high pH level (i.e., alkaline-treated plants) (**Table 2**). Averaged over nutrient solution treatment, the highest values of lettuce fresh yield were recorded in the Tablet + PH application treatment, followed by the Tablet application, whereas the lowest values were observed in the untreated lettuce plants. Moreover, under saline, alkaline and standard nutrient solution conditions, the percentage of yield increase in comparison to the control (i.e., no application) was higher with Tablet + PH application (62, 40, and 43%, respectively) than with the microbial biostimulant tablet (46, 25, and 18%, respectively) (**Table 2**). The lowest root dry weight was recorded in untreated lettuce plants irrigated with both saline and alkaline nutrient solutions (**Table 2**).

**Table 2:** Analysis of variance and mean comparisons for shoot fresh weight, dry weight of shoots and roots, total root length, average root diameter, and total root surface of lettuce plants grown with different nutrient solutions and biostimulants.

Source of variance	Shoot fresh weight (g/plant)	Shoot dry weight (g plant^-1^)	Root dry weight (g plant^-1^)	Total root length (m plant^-1^)	Root diameter (mm)	Total root surface (cm^2^ plant^-1^)
Nutrient solution (N)	^∗∗∗^	^∗∗^	^∗^	^∗∗∗^	ns	^∗∗^
Biostimulant (B)	^∗∗∗^	^∗∗∗^	^∗∗^	^∗∗^	ns	^∗^
N × B	ns	ns	^∗^	ns	ns	ns
Nutrient solution						
Standard	175.4ˆa	7.81ˆa	1.86	531.4ˆa	0.29	48.4ˆa
Alkaline	161.6ˆa	7.87ˆa	1.79	484.1ˆb	0.30	44.5ˆab
Saline	119.4ˆb	7.02ˆb	1.55	405.1ˆc	0.28	37.0ˆb
Biostimulant						
No application	122.2ˆc	6.03ˆb	1.47	436.8ˆc	0.28	40.5ˆc
Tablet	155.2ˆb	8.06ˆa	1.75	470.4ˆb	0.29	42.7ˆb
Tablet + PH	179.2ˆa	8.62ˆa	1.98	510.1ˆa	0.29	46.5ˆa
N × B						
Standard solution without biostimulant	145.8	5.93	1.63ˆbc	494.1	0.29	45.8
Standard solution + Tablet	171.5	8.45	1.84ˆab	538.5	0.29	48.7
Standard solution + Tablet + PH	209.1	9.05	2.12ˆa	561.6	0.30	50.6
Alkaline solution without biostimulant	132.7	6.53	1.48ˆcd	460.4	0.29	43.2
Alkaline solution + Tablet	166.0	8.37	1.81ˆab	472.0	0.30	43.3
Alkaline solution + Tablet + PH	186.3	8.72	2.08ˆa	519.8	0.30	47.1
Saline solution without biostimulant	88.0	5.62	1.31ˆd	355.9	0.27	32.6
Saline solution + Tablet	128.0	7.36	1.61ˆbc	400.8	0.29	36.1
Saline solution + Tablet + PH	142.3	8.09	1.74ˆbc	458.6	0.28	42.4

*ns, ^∗^, ^∗∗^, ^∗∗∗^ Non-significant or significant at *P* ≤ 0.05, 0.01, and 0.001, respectively. Different letters within each column indicate significant differences according to Duncan’s multiple-range test (*P* = 0.05). Tablet = microbial-based biostimulant ‘Click Horto’ containing 200 spores/tablet of *R. irregularis* BEG72 plus 4.5 × 10^*7*^ CFU/tablet of *T. atroviride* MUCL45632; PH = plant derived-protein hydrolysate ‘Trainer.’*

Neither nutrient solution nor biostimulant treatment had a significant effect on root diameter (average 0.29 mm). The total root length and total root surface were negatively affected by salt stress treatment, with a significant decrease in both root parameters (**Table 2**). Averaged over nutrient solution, the total root length in Tablet + PH treatment was higher than those recorded in Tablet-treated and untreated plants by 8.5 and 16.8%, respectively. Similarly, to total root length, the Tablet + PH treatment positively affected the total root surface, which was higher by 9.4 and 15.3% compared to microbial biostimulant and untreated plants, respectively.

### Leaf Mineral Composition

The macronutrient, sodium and iron concentration as a function of nutrient solution composition and biostimulant application is displayed in **Table 3**. The N concentration in leaves was influenced by nutrient solution composition and biostimulant treatment with no significant N × B interaction (**Table 3**). Leaf concentration of N decreased in plants irrigated with saline solution treatments in comparison with the control treatment (**Table 3**). Moreover, highest N concentration was recorded in the biostimulant treatment under both Tablet and Tablet + PH application compared to untreated lettuce plants (**Table 3**).

**Table 3:** Analysis of variance and mean comparisons for mineral composition of leaves from lettuce plants grown with different nutrient solutions and biostimulants.

Source of variance	N (g kg^-1^ d.wt.)	P (g kg^-1^ d.wt.)	K (g kg^-1^ d.wt.)	Na (g kg^-1^ d.wt.)	Fe (mg kg^-1^ d.wt.)
Nutrient solution (N)	^∗^	^∗∗^	^∗∗∗^	^∗∗∗^	^∗∗^
Biostimulant (B)	^∗∗^	^∗∗^	^∗^	^∗^	^∗^
N × B	ns	^∗∗^	^∗∗^	^∗∗∗^	^∗∗^
Nutrient solution					
Standard	40.3ˆa	7.1	33.0	2.9	45.5
Alkaline	38.5ˆab	5.5	32.9	3.3	39.7
Saline	36.3ˆb	6.2	29.2	11.3	41.8
Biostimulant					
No application	35.3ˆb	5.7	29.7	7.6	40.2
Tablet	39.1ˆa	6.5	32.4	4.9	42.7
Tablet + PH	40.6ˆa	6.6	33.0	5.0	44.0
N × B					
Standard solution without biostimulant	38.9	6.6ˆb	32.6ˆbc	3.2ˆc	45.6ˆab
Standard solution + Tablet	40.9	7.3ˆa	33.0ˆab	2.7ˆc	44.9ˆab
Standard solution + Tablet + PH	41.0	7.5ˆa	33.5ˆab	2.9ˆc	45.9ˆa
Alkaline solution without biostimulant	35.5	4.7ˆd	30.7ˆd	4.1ˆc	35.9ˆf
Alkaline solution + Tablet	38.6	5.9ˆc	33.9ˆab	2.8ˆc	40.8ˆde
Alkaline solution + Tablet + PH	41.3	6.0ˆbc	34.2ˆa	3.1ˆc	42.3ˆcd
Saline solution without biostimulant	31.6	5.9ˆc	25.8ˆe	15.6ˆa	39.0ˆe
Saline solution + Tablet	37.8	6.4ˆb	30.3ˆd	9.3ˆb	42.5ˆcd
Saline solution + Tablet + PH	39.5	6.3ˆb	31.4ˆcd	8.9ˆb	43.9ˆbc

*ns, ^∗^, ^∗∗^, ^∗∗∗^ Non-significant or significant at *P* ≤ 0.05, 0.01, and 0.001, respectively. Different letters within each column indicate significant differences according to Duncan’s multiple-range test (*P* = 0.05). Tablet = microbial-based biostimulant ‘Click Horto’ containing 200 spores/tablet of *R. irregularis* BEG72 plus 4.5 × 10^*7*^ CFU/tablet of *T. atroviride* MUCL45632; PH = plant derived-protein hydrolysate ‘Trainer.’*

The P, K, Na, and Fe concentrations in lettuce leaves were highly affected by N × B interaction (**Table 3**). For instance, under alkaline conditions, concentrations of P and K in Tablet and Tablet + PH treated plants were higher than those in untreated plants (**Table 3**). Similarly, under NaCl conditions, plants coming from the Tablet and Tablet + PH treatments had the highest P and K concentration in leaves in comparison to untreated plants.

The lowest Na concentration in leaf tissue was observed under both untreated and treated lettuce plants irrigated with alkaline and standard solution, whereas the highest values were recorded in untreated lettuce plants under saline conditions (**Table 3**). Moreover, under saline conditions, the application of Tablet or Tablet + PH reduced significantly the leaf Na concentration in comparison with untreated plants (**Table 3**). The reduction of Fe in leaf tissue of plants treated with NaHCO_3_, with respect to standard solution, was significantly lower in plants treated with biostimulant in comparison to that of untreated plants. A similar trend was also observed under NaCl conditions where the leaf Fe concentration reductions in comparison to basic nutrient solution were clearly lower in plants treated with Tablet and Tablet + PH in comparison to untreated plants (**Table 3**).

### SPAD Index and Chlorophyll Fluorescence

The SPAD index of lettuce at 13, 20, and 36 DAT as well as the maximum quantum use efficiency of PSII in dark-adapted state at 13 and 36 DAT were significantly affected by nutrient solution and biostimulant treatment, with no interaction of these two factors (**Table 4**). Irrespective of the biostimulant treatment, increasing the NaCl concentration from 1 to 25 mM, decreased the SPAD index at 13, 20, and 36 DAT, whereas under alkaline conditions this reduction was only recorded at 13 DAT (**Table 4**). When averaged over nutrient solution, the highest values of SPAD at 13 and 36 DAT were observed in Tablet + PH-treated plants, followed by Tablet treatment, whereas the lowest values were recorded in the untreated lettuce plants.

**Table 4:** Analysis of variance and mean comparisons for SPAD index and maximum quantum use efficiency PSII in dark-adapted stage of leaves at different days after transplanting (DAT) from lettuce plants grown with different nutrient solutions and biostimulants.

Source of variance	SPAD index			Maximum quantum use efficiency PSII (*F*_v_/*F*_m_)		
	13 DAT	20 DAT	36 DAT	13 DAT	20 DAT	36 DAT
Nutrient solution (N)	^∗∗^	^∗∗∗^	^∗∗∗^	^∗∗∗^	^∗∗∗^	^∗∗^
Biostimulant (B)	^∗∗∗^	^∗^	^∗^	^∗∗^	ns	^∗^
N × B	ns	ns	ns	ns	^∗^	ns
Nutrient solution						
Standard	34.9^a^	36.4^a^	34.4^a^	0.79^a^	0.87	0.86^a^
Alkaline	31.2^b^	35.6^a^	33.7^a^	0.78^a^	0.86	0.86^a^
Saline	31.0^b^	31.4^b^	28.8^b^	0.74^b^	0.81	0.81^b^
Biostimulant						
No application	28.3^c^	33.2^b^	30.0^c^	0.75^b^	0.84	0.83^b^
Tablet	33.4^b^	34.4^ab^	32.8^b^	0.78^a^	0.85	0.84^ab^
Tablet + PH	35.4^a^	35.8^a^	34.2^a^	0.79^a^	0.85	0.85^a^
N × B						
Standard solution without biostimulant	29.4	35.4	33.9	0.77	0.86^a^	0.85
Standard solution + Tablet	36.9	36.0	34.2	0.80	0.87^a^	0.86
Standard solution + Tablet + PH	38.5	37.8	35.2	0.81	0.87^a^	0.87
Alkaline solution without biostimulant	28.7	34.6	31.1	0.76	0.86^a^	0.85
Alkaline solution + Tablet	31.4	35.3	34.6	0.78	0.86^a^	0.86
Alkaline solution + Tablet + PH	33.5	37.0	35.5	0.79	0.87^a^	0.86
Saline solution without biostimulant	26.8	29.6	25.1	0.71	0.79^b^	0.78
Saline solution + Tablet	32.0	31.9	29.5	0.75	0.83^ab^	0.81
Saline solution + Tablet + PH	34.1	32.7	31.9	0.77	0.82^ab^	0.83

*ns, ^∗^, ^∗∗^, ^∗∗∗^ Non-significant or significant at *P* ≤ 0.05, 0.01, and 0.001, respectively. Different letters within each column indicate significant differences according to Duncan’s multiple-range test (*P* = 0.05). Tablet = microbial-based biostimulant ‘Click Horto’ containing 200 spores/tablet of *R. irregularis* BEG72 plus 4.5 × 10^*7*^ CFU/tablet of *T. atroviride* MUCL45632; PH = plant derived-protein hydrolysate ‘Trainer.’*

The efficiency of the PSII in dark-adapted leaves measured as the *F*_v_/*F*_m_ ratio was decreased in NaCl-treated plants at 13 and 36 DAT regardless of the biostimulant treatment, whereas NaHCO_3_ had no effect (**Table 4**). At 20 DAT, the efficiency of the PSII was decreased only in untreated lettuce plants grown under saline conditions. Finally, irrespective of the nutrient solution, the highest *F*_v_/*F*_m_ ratio at 13 and 36 DAT were recorded in biostimulant treatment under both Tablet and Tablet + PH application compared to untreated lettuce plants.

### Antioxidant Enzyme Activities and Proline Content

Stress response of biostimulant-treated and untreated lettuce plants exposed to salinity and alkalinity conditions was evaluated by analyzing changes in capacity of two important antioxidative enzymes (CAT and GPX) on expanded leaves. In the present study, the changes in antioxidative enzyme activities were mainly due to the biostimulant treatment and not to the nutrient solution composition (**Table 5**). Irrespective of nutrient solution, the highest CAT and GPX activities in leaf crude extract were observed in both biostimulant treatments compared to untreated lettuce plants. The differences between the two biostimulant treatments were not significant.

**Table 5:** Analysis of variance and mean comparisons for catalase, guaiacol peroxidase, and proline content of leaves from lettuce plants grown with different nutrient solutions and biostimulants.

Source of variance	Catalase	Guaiacol peroxidase	Proline
	(mmol mg^-1^ protein min^-1^)	(mmol mg^-1^ protein min^-1^)	(μg g^-1^ f. wt.)
Nutrient solution (N)	ns	ns	^∗∗^
Biostimulant (B)	^∗∗^	^∗^	^∗^
N × B	ns	ns	ns
Nutrient solution			
Standard	94.0	87.0	22.4ˆb
Alkaline	105.4	72.9	24.6ˆb
Saline	99.3	90.9	69.5ˆa
Biostimulant			
No application	56.1ˆb	60.0ˆb	22.5ˆc
Tablet	118.1ˆa	90.9ˆa	44.5ˆb
Tablet + PH	124.5ˆa	100.0ˆa	49.6ˆa
N × B			
Standard solution without biostimulant	50.4	68.7	12.1
Standard solution + Tablet	111.6	91.0	25.2
Standard solution + Tablet + PH	120.1	101.4	29.9
Alkaline solution without biostimulant	59.2	52.2	18.4
Alkaline solution + Tablet	126.8	81.6	26.9
Alkaline solution + Tablet + PH	130.3	85.0	28.5
Saline solution without biostimulant	58.8	59.0	36.9
Saline solution + Tablet	116.0	100.2	81.4
Saline solution + Tablet + PH	123.2	113.6	90.3

*ns, ^∗^, ^∗∗^ Non-significant or significant at *P* ≤ 0.05, and 0.01, respectively. Different letters within each column indicate significant differences according to Duncan’s multiple-range test (*P* = 0.05). Tablet = microbial-based biostimulant ‘Click Horto’ containing 200 spores/tablet of *R. irregularis* BEG72 plus 4.5 × 10^*7*^ CFU/tablet of *T. atroviride* MUCL45632; PH = plant derived-protein hydrolysate ‘Trainer.’*

Analysis of proline in leaves revealed marked differences between the nutrient solution and biostimulant treatments (**Table 5**). The leaf proline content was increased substantially by 210% in NaCl-treated plants, whereas no significant effect was recorded with NaHCO_3_ (**Table 5**). Consistent with the antioxidant activities, the proline content increased significantly by 97.8 and 120.4% when lettuce was supplied with Tablet and Tablet + PH, respectively, in comparison to untreated plants.

## Discussion

Under adverse chemical soil conditions such as alkalinity and salinity, plant growth inhibition and consequently yield reduction are induced by different physiological and biochemical alterations (Colla et al., 2008, 2010; Cardarelli et al., 2010). However, the severity of crop productivity losses may change in relation to several interacting parameters, including the phenological stage, environmental conditions and the magnitude of the stress experienced over time (i.e., salt concentration and time of exposure) (Cartmill et al., 2008; Munns and Tester, 2008). In the present experiment, the decrease in biomass production under alkali stress was not significant, whereas NaCl application had negative influence on lettuce fresh yield, shoot and root dry weight. The higher degree of biomass loss under salinity stress compared to alkalinity suggests that the 25 mM of NaCl in the nutrient solution had a greater negative impact on lettuce performance than alkaline conditions (pH 8.1). The detrimental effects of salinity not only affect the lettuce plant but also the AM symbiosis. In fact, the lowest percentage of AM infection was observed at 25 mM NaCl indicating the adverse effects of salt stress on germination of spore, hyphal development and growth as well as on the production of arbuscules (Miransari, 2010). The stunted crop growth, root morphology and yield observed under saline nutrient solution has been demonstrated in greenhouse studies on mini-watermelon and pepper (Colla et al., 2006a,b), cucumber (Colla et al., 2012, 2013a), tomato (Savvas et al., 2011), melon and zucchini squash (Rouphael et al., 2012, 2017) grown hydroponically.

Reduced lettuce shoot weight under saline treatment could be attributed (i) to osmotic stress generated by increased osmotic potential in the growth medium and (ii) to salinity-induced nutritional imbalance related to excessive ion uptake (e.g., Na^+^ and/or Cl^-^) within the plant (Munns, 2005). In this study, macro- and micronutrient and sodium concentrations recorded in leaf tissues indicate that lettuce entered the salt-specific effect stage, since increasing NaCl from 1 to 25 mM in the nutrient solution increased the Na uptake and accumulation (+290%) leading to nutritional disorders especially for N, P, K, and Fe. The nutritional imbalance of plants has been attributed to several mechanisms including osmotic effects of salts and also to the competition between Na^+^ and K^+^ uptake in roots leading to extreme ratio of Na^+^/K^+^ (Grattan and Grieve, 1999; Munns, 2005). As a result, lettuce plant become more sensitive to specific ion injury as well as to mineral deficiencies resulting in stunted growth and productivity loss. Concerning the effect of alkaline solution on nutrient uptake, our results indicated that alkali stress exerted no significant effect on N, K, and Na concentrations in lettuce leaves. This result indicated that lettuce is quite tolerant to alkalinity. Furthermore, it has been reported in many plant species that an alkaline pH in the nutrient solution or the rhizosphere is a key factor limiting P and Fe availability for plants, with higher pH reducing availability (Alhendawi et al., 1997; Marschner, 2012). This could explain why P and Fe concentrations in lettuce leaves were negatively correlated with the application of NaHCO_3_ providing a reduction of 22.5 and 12.7%, respectively, compared to the standard solution.

However, when lettuce plants were treated with microbial biostimulant (Tablet) and especially Tablet + PH the extent of fresh marketable yield and biomass production suppression was significantly reduced compared to untreated plants. The co-inoculation of *R. intraradices* BEG72 and *T. atroviride* MUCL45632 markedly improved the lettuce fresh yield (+27%). A presumed mechanism involved in the stimulation of crop performance after co-inoculation with beneficial microorganisms might be the improvement of mineral nutrient availability and plant uptake. Nutrient uptake is a primary factor for the maintenance of homeostasis and plant growth under edaphic adversities (Marschner, 2012). In the present study, both biostimulant treatments reduced Na uptake of plants and increased the K uptake, compared to untreated plants under salt stress, thus increasing K/Na ratio. The capacity to maintain a high K/Na ratio in the shoot tissue constitutes an important salt tolerance indicator (Møller et al., 2009). The greatest K accumulation, and the reduced Na concentration in the microbial treated lettuce, may have helped the plants toward maintaining the osmotic potential in their cells and also preventing accumulation of cellular Na to a toxic concentration. Moreover, biostimulant-treated and untreated plants diverged in their tissue nutrient concentration in relation to alkalinity. Lettuce plants supplied with Tablet and Tablet + PH had a strong capacity to accumulate Fe in leaf tissue under alkaline conditions and were also able to maintain a better plant nutritional status (higher P and K concentration). Our results are consistent with several previous studies (Cartmill et al., 2008; Colla et al., 2008; Cardarelli et al., 2010; Rouphael et al., 2010; Ahmad et al., 2015) which showed an improvement in the uptake of some macro- and microelements under salinity and alkalinity conditions, when plants were inoculated with AM fungi or *Trichoderma* spp..

The enhanced nutritional status has often been associated with the ability of mycorrhiza and/or *Trichoderma* to alter the root system architecture (i.e., morphology), yielding more extensive absorbing area, which may be considered a mechanism of abiotic stress tolerance (Kohler et al., 2009; Wu et al., 2013). This was the case in the current experiment, since the total root length and surface increased significantly by 16.8 and 9.4% when lettuce plants were supplied with Tablet + PH application and by 7.7 and 5.5% with microbial based biostimulant, compared to untreated plants. The increase of root length and density induced by AM fungi may be attributed to several growth improving mechanisms like (i) stimulation of root auxin production, (ii) endogenous balance of cytokinin to gibberellins, (iii) regulated metabolism of endogenous polyamine, and (iv) better nutritional status in AM host plants (Ludwig-Müller and Güther, 2007; Wu et al., 2013; Rouphael et al., 2015b). Furthermore, the production of hormones like auxin or analogs, small peptides, siderophores, volatiles and enzymes by the fungal mycelium are also considered mechanisms by which strains of *Trichoderma* can promote root branching and nutrient uptake, thereby boosting plant growth and development under environmental stress (Doni et al., 2014; Colla et al., 2015a; López-Bucio et al., 2015).

Our results further demonstrated that the combination of microbial tablet with foliar application of PH synergistically increased the marketable fresh yield by 15.5 and 46.7% compared to the Tablet-treated and untreated plants, respectively. Several studies showed the positive effects exerted by PHs application alone in reducing losses in production caused by unfavorable soil conditions including salinity (Ertani et al., 2013; Lucini et al., 2015) and alkalinity (Cerdán et al., 2009). These authors concluded that the better crop performance of PHs-treated plants has been attributed to (i) higher nutrient uptake, (ii) better root system architecture, (iii) osmotic adjustment, and (iv) increase of several secondary metabolites (e.g., flavonoids, terpenes, and glucosinolates). Interestingly, in our study, a synergistic action on lettuce performance was found when beneficial microorganisms and PH were applied together.

The different effects between the biostimulant applications on agronomical parameters should be also reflected at physiological and biochemical levels. This was the case in the current study, since the main physiological changes, in particular chlorophyll content and fluorescence induced by biostimulant applications, were recorded in the leaves of lettuce plant. When averaged over nutrient solution, the SPAD index dropped sharply (at 13, and 36 DAT) in untreated lettuce compared to both biostimulant-treated plants. This suggests the occurrence of chlorophyll breakdown, likely due to the detrimental effects of ROS on chloroplasts (Rouphael et al., 2015b). Contrarily, the biostimulant-treated plants were able to maintain a higher SPAD index irrespective of nutrient solution treatments, thus exhibiting the highest marketable fresh yield. Overall, the beneficial effect of Tablet + PH in protecting chlorophyll degradation (highest SPAD index) may be attributed to an improvement in the uptake of bivalent cations principally Mg^2+^ and Fe^2+^, that are required for chlorophyll biosynthesis (Sheng et al., 2008). Our results are in agreement with the finding of several authors (Colla et al., 2008, 2015a; Ertani et al., 2013; Rouphael et al., 2010, 2015b; Ahmad et al., 2015), who demonstrated that application of AMF, *Trichoderma* or PHs were able to restore the chlorophyll content to acceptable levels under saline and alkaline conditions in comparison to untreated plants. On the other hand, the reduction of SPAD index was accompanied by a decline of the maximum quantum yield of photosystem (PS) II (*F*_v_/*F*_m_) in untreated lettuce plants. In fact, when averaged over nutrient solution, *F*_v_/*F*_m_ ratio was significantly reduced at 13, 20, and 36 DAT in untreated plants, indicating the occurrence of photoinhibition and this could be a consequence of stress damage to the PSII (Demmig-Adams and Adams, 1992). However, maintenance of high *F*_v_/*F*_m_ in both biostimulant treatments may have been crucial to delay photoinhibition and to guarantee a better functioning of the photosynthetic apparatus, thus increasing the final yield of lettuce.

An important system of protection against abiotic stress includes the synthesis of osmolytes, such as proline, which play an important role by protecting cell membranes and proteins from damage as well as quenching ROS (Kaur and Asthir, 2015). In our study, The Tablet + PH treated plants showed maximum accumulation of proline as compared to other treatments contributing to a better osmotic adjustment and providing a greater protection to the cells from salt and alkali stress. Thus, the higher accumulation of proline in Tablet + PH combination supports the observed higher salt/alkali tolerance in biostimulant-treated than untreated plants. Moreover, antioxidant enzymes, such as CAT and GPX responded differently under different biostimulant applications. The activities of GPX and especially CAT, which is the major enzyme responsible for intracellular hydrogen peroxide (H_2_O_2_) degradation (Vanacker et al., 1998), was significantly higher in biostimulant-treated than in untreated plants. Overall, our findings suggest that the combined application Tablet + PH induce the activation of both proline and antioxidant enzyme activities as a strategy against oxidative damage in lettuce plants.

## Conclusion

**FIGURE 1:**
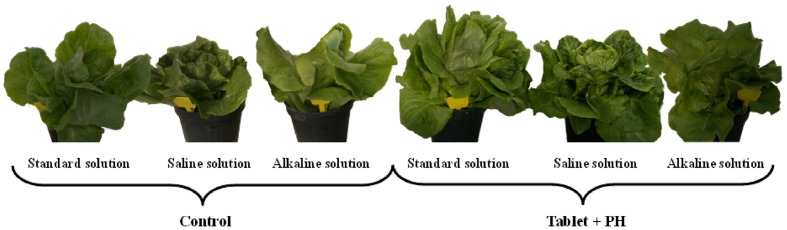
**Effects of nutrient solution and biostimulant application [no application of biostimulants (Control); microbial-based biostimulant ‘Click Horto’ (Tablet) plus the plant derived-protein hydrolysate ‘Trainer’ (PH)] on lettuce plants**.

Abiotic stresses, such as alkalinity and salinity, are the major environmental threats limiting crop productivity worldwide. Thus, it is important to investigate whether the use of plant biostimulants may represent a potential approach to decrease the deleterious effects of saline and alkaline stress in an important commercial crop such as lettuce. Our findings indicate that in both biostimulant-treated and untreated plants, the shoot fresh yield and dry biomass decreased in response to increasing salinity, with no detrimental effect recorded with alkaline solution. The percentage of yield increase in comparison to the control was markedly higher in Tablet + PH application (**Figure 1**) followed by the tablet based biostimulant and finally in untreated plants. The application of biostimulant as Tablet or Tablet + PH was capable of maintaining higher chlorophyll content and photochemical activity of PSII, and a better nutritional status in the leaf tissue. The results of our experiment were able to verify our hypothesis, that combined application of microbial-based biostimulant and PH, was more effective than microbial biostimulant application alone to mitigate the negative effect of stress on the growth of lettuce. The improved crop performance of Tablet + PH application was attributed to a better root system architecture, an improved chlorophyll synthesis and an increase in proline accumulation.

## Author Contributions

YR performed the experiment with agronomic and physiological analysis, and he was involved in data analysis, results interpretation, and writing the manuscript. MC performed the mineral analysis and enzyme assays, and she collaborated in manuscript preparation. PB performed the microbial analysis, and he collaborated in manuscript preparation. GC defined the scientific hypothesis, set up the experimental protocol, made the statistical analysis of experimental data, and he was involved in the manuscript preparation.

## Conflict of Interest Statement

The research was partially supported by Italpollina Company in the frame of the agreement between the Italpollina and the Dept. DAFNE, Tuscia University.
